# A Rare Case of Complete Agenesis of Dorsal Pancreas

**DOI:** 10.5005/jp-journals-10018-1245

**Published:** 2017-09-29

**Authors:** Atul Jain, Malwinder Singh, Subhajeet Dey, Ankit Kaura, Gaurav Diwakar

**Affiliations:** 1 Department of Surgery, ESI Post Graduate Institute of Medical Sciences & Research, ESI Hospital, Basaidarapur, New Delhi, India

**Keywords:** Agenesis of dorsal pancreas, Developmental failure, Diabetes, Magnetic resonance cholangiopancreatography.

## Abstract

Agenesis of dorsal pancreas (ADP) is an extremely rare congenital anomaly that results from defective development of pancreas. Most ADP patients are asymptomatic; if symptomatic, they present with epigastric pain. About half of affected individuals develop diabetes mellitus (DM), resulting from reduced islet cell mass secondary to the absence of endocrine structures. Being very rare, it is generally not kept in mind while dealing these cases and are not suspected until imaging investigations are not done. In our case study, ADP was diagnosed during evaluation of the patient for recurrent pain abdomen and generalized weakness.

**How to cite this article:** Jain A, Singh M, Dey S, Kaura A, Diwakar G. A Rare Case of Complete Agenesis of Dorsal Pancreas. Euroasian J Hepato-Gastroenterol 2017;7(2):183-184.

## INTRODUCTION

Agenesis of dorsal pancreas is an extremely rare congenital anomaly that results from defective pancreas development. The first case was reported in 1911 during an autopsy and around 100 cases have been reported since then.^1,2^ Majority of the cases are diagnosed incidentally during the workup for an unrelated abdominal pain. Here we present a case of ADP diagnosed during workup of pain abdomen and DM.

## CASE REPORT

An otherwise healthy 37-year-old female presented to the outpatient department with complaint of recurrent upper abdominal pain, fatigue, dizziness, and pain in lower extremities for past few months. General physical and systemic examination was normal.

**Fig. 1:**
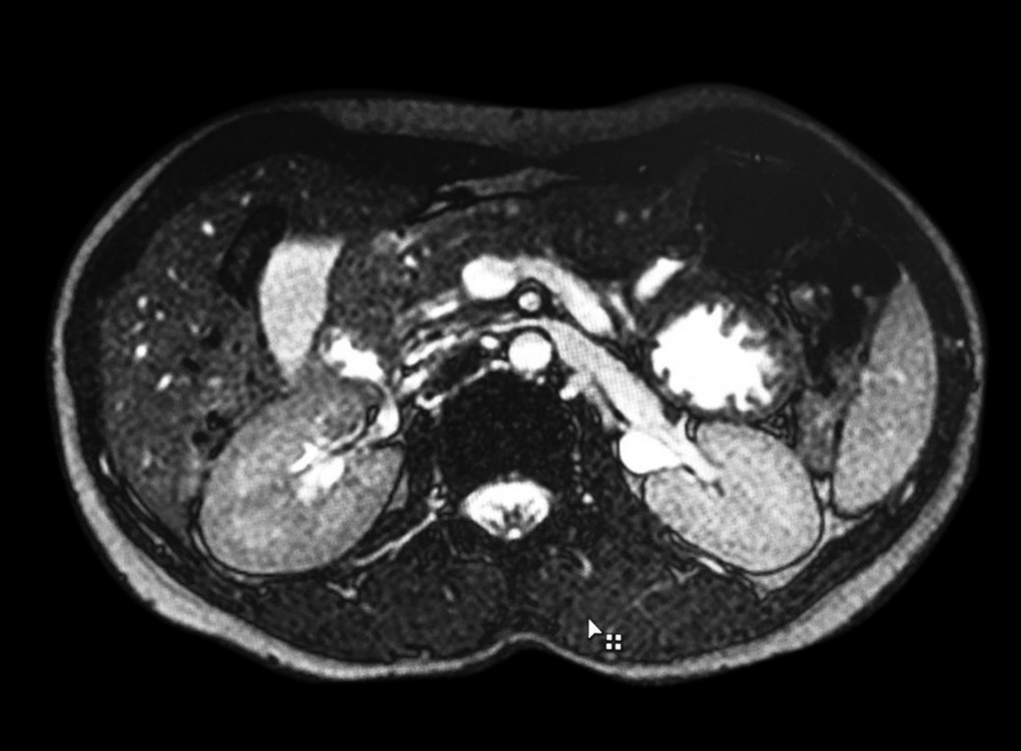
MRCP showing agenesis of dorsal pancreas

**Figs 2A and B:**
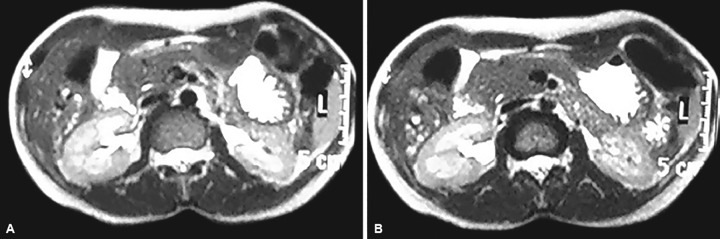
MRCP showing agenesis of dorsal pancreas and pancreatic duct

Routine blood investigations revealed high blood sugar level (454 mg/dL) and she also had glucosuria. Patient was evaluated for recurrent abdominal pain and subsequently workup for DM was done. Ultrasound abdomen was done, in which only head of pancreas was seen and distal pancreas and duct were not visible. Magnetic resonance cholangiopancreatography (MRCP) was subsequently done in which distal body and tail of pancreas were not visible and diagnosis of ADP was made (Figs 1 and 2). Patient symptomatically improved after dietary modification and treatment of DM was started.

## DISCUSSION

Abnormal embryogenesis can lead to developmental failure of the dorsal pancreas, resulting in complete ADP.^3^ Agenesis of the ventral pancreas and complete agenesis of the pancreas are incompatible with life.^4^

The pancreas is formed by ventral and dorsal endodermal buds. The ventral bud gives rise to the major part of the head and uncinate process which drains through Wirsung duct. The dorsal bud forms the upper part of the head, body, and tail of the pancreas which drains through the Santorini duct.^5^ The cause of ADP is currently not well understood. Familial transmission has been reported in the literature.^6^ Differential diagnosis of ADP includes pancreas divisum (failure of the ventral and dorsal pancreatic ducts to fuse), pseudo-agenesis (atrophy of the body and the tail of the pancreas secondary to chronic pancreatitis and sparing of the pancreatic head), and congenital short pancreas.^7^

Most ADP patients are asymptomatic; if symptomatic, they present with epigastric pain. About half of the affected individuals develop DM, resulting from reduced islet cell mass secondary to the absence of endocrine structures, which are normally predominantly located in the body and tail of the pancreas. A connection to pancreatitis has also been proposed. Potential mechanisms include sphincter of Oddi dysfunction, compensatory enzyme hypersecretion, hypertrophy of the ventral gland, and higher pancreatic duct pressures.^8^ A case of pancreatic adenocarcinoma associated with dorsal agenesis has also been reported.^9^

The most frequent imaging techniques used ultrasound and computed tomography scan, but they are suggestive of ADP. Further confirmatory imaging techniques are magnetic resonance imaging, endoscopic retrograde cholangiopancreatography (ERCP), MRCP, and, recently, endoscopic ultrasound.^10^ Moreover, ERCP or MRCP can define the anatomy of the pancreatic ducts more precisely.^11^ Treatment is usually symptomatic and supportive.

Being very rare, it is generally not kept in mind while dealing with such presentations and not suspected until imaging investigations are done. In our case study, ADP was diagnosed during evaluation of the patient for pain abdomen and generalized weakness.

## CONCLUSION

In patients with unexplained pain abdomen and with constitutional symptoms, a thorough evaluation should be done and anomalies such as, ADP should be kept in mind.
